# Retraction: Molecular Cloning and Docking of *speB* Gene Encoding Cysteine Protease With Antibiotic Interaction in *Streptococcus pyogenes* NBMKU12 From the Clinical Isolates

**DOI:** 10.3389/fmicb.2018.03309

**Published:** 2018-12-21

**Authors:** Frontiers Editorial Office

The Journal and Chief Editors retract the August 2018 article cited above. Following publication, concerns were brought to the attention of the publisher regarding the validity of the article's findings. The conclusion from the assessment of the Specialty and Field Chief Editor is that the article reports unsupported conclusions.

The article is retracted in the absence of the authors' agreement.

